# A reactive oxygen species–related signature to predict prognosis and aid immunotherapy in clear cell renal cell carcinoma

**DOI:** 10.3389/fonc.2023.1202151

**Published:** 2023-07-11

**Authors:** Hongxiang Liu, Yong Luo, Shankun Zhao, Jing Tan, Minjian Chen, Xihai Liu, Jianheng Ye, Shanghua Cai, Yulin Deng, Jinchuang Li, Huichan He, Xin Zhang, Weide Zhong

**Affiliations:** ^1^ School of Medicine, Jinan University, Guangzhou, China; ^2^ Department of Urology, The First People’s Hospital of Zhaoqing, Zhaoqing, China; ^3^ Department of Urology, The Second People’s Hospital of Foshan, Affiliated Foshan Hospital of Southern Medical University, Foshan, China; ^4^ Department of Urology, Taizhou Central Hospital (Taizhou University Hospital), Taizhou, China; ^5^ Department of Pediatrics, The First People’s Hospital of Zhaoqing, Zhaoqing, China; ^6^ Department of Urology, Guangdong Key Laboratory of Clinical Molecular Medicine and Diagnostics, Guangzhou First People’s Hospital, School of Medicine, South China University of Technology, Guangzhou, China; ^7^ Urology Key Laboratory of Guangdong Province, The First Affiliated Hospital of Guangzhou Medical University, Guangzhou Medical University, Guangzhou, China; ^8^ Guangzhou Medical University, Guangzhou Laboratory, Guangzhou, China; ^9^ Department of Pathology, The Second People’s Hospital of Foshan, Affiliated Foshan Hospital of Southern Medical University, Foshan, China; ^10^ Macau Institute for Applied Research in Medicine and Health, Macau University of Science and Technology, Macau, Macao SAR, China

**Keywords:** clear cell renal cell carcinoma, reactive oxygen species, prognosis, immune infiltrates, immunotherapy

## Abstract

**Background:**

Clear cell renal cell carcinoma (ccRCC) is a malignant disease containing tumor-infiltrating lymphocytes. Reactive oxygen species (ROS) are present in the tumor microenvironment and are strongly associated with cancer development. Nevertheless, the role of ROS-related genes in ccRCC remains unclear.

**Methods:**

We describe the expression patterns of ROS-related genes in ccRCC from The Cancer Genome Atlas and their alterations in genetics and transcription. An ROS-related gene signature was constructed and verified in three datasets and immunohistochemical staining (IHC) analysis. The immune characteristics of the two risk groups divided by the signature were clarified. The sensitivity to immunotherapy and targeted therapy was investigated.

**Results:**

Our signature was constructed on the basis of glutamate-cysteine ligase modifier subunit (GCLM), interaction protein for cytohesin exchange factors 1 (ICEF1), methionine sulfoxide reductase A (MsrA), and strawberry notch homolog 2 (SBNO2) genes. More importantly, protein expression levels of GCLM, MsrA, and SBNO2 were detected by IHC in our own ccRCC samples. The high-risk group of patients with ccRCC suffered lower overall survival rates. As an independent predictor of prognosis, our signature exhibited a strong association with clinicopathological features. An accurate nomogram for improving the clinical applicability of our signature was constructed. Gene Ontology and Kyoto Encyclopedia of Genes and Genomes analyses showed that the signature was closely related to immune response, immune activation, and immune pathways. The comprehensive results revealed that the high-risk group was associated with high infiltration of regulatory T cells and CD8+ T cells and more benefited from targeted therapy. In addition, immunotherapy had better therapeutic effects in the high-risk group.

**Conclusion:**

Our signature paved the way for assessing prognosis and developing more effective strategies of immunotherapy and targeted therapy in ccRCC.

## 1 Introduction

Renal cell carcinoma (RCC) is the third most common malignant tumor of the genitourinary system, which afflicted more than 430,000 people and caused approximately 180,000 deaths in 2020 (1, 2). Clear cell RCC (ccRCC) is the predominant pathological type, comprising more than 80% of RCC (3). At present, radical surgery is the first choice for early-stage ccRCC. However, local recurrence and distant metastasis will still occur even after radical nephrectomy, necessitating further understanding the molecular mechanism of ccRCC to determine a new approach or biomarker that can accurately predict prognosis and guide clinical treatment (4, 5).

Unlike other genitourinary malignancies, ccRCC is highly intrinsically insensitive to chemotherapy and radiation therapy (6, 7). It has inspired the discovery of a range of alternative therapies, including immunotherapy and targeted therapy. As a highly immunogenic tumor, ccRCC exhibits unparalleled levels of immune infiltration compared to other types of cancers, which has stimulated the exploration of immunotherapy in ccRCC (8, 9). Immune checkpoint inhibitors (ICIs) have made significant advancements and demonstrated evident efficacy in patients with ccRCC, regardless of whether the patients had been treated before (10). Motzer et al. (11) found that patients with advanced ccRCC showed an amazing response rate of 25% after receiving the nivolumab, programmed cell death protein 1 (PD-1) inhibitor. However, some patients still responded poorly to immunotherapy and even metastasized during immunotherapy (12).

Reactive oxygen species (ROS) are oxygen-containing molecules with high reactivity, a by-product of cell metabolism, and are mainly produced in mitochondria. Elevated ROS is observed in almost all cancers, and ROS has been instrumental in driving the biological progression of cancers (13). Meanwhile, as an important cell signaling molecule, ROS dynamically and diversely affected many aspects of tumor development and progression. ROS could initiate cancer angiogenesis and also stimulate the cancer cell survival signal cascade to promote cancer cell metastasis, progression, and adaptation to hypoxia. High concentrations of ROS can promote anti-tumor signals and trigger cancer cell death induced by oxidative stress (14). Immune cells can specifically recognize and kill tumor cells. Meanwhile, tumor cells disrupt immune surveillance by harming immune cells to block the immune response (15). In this dynamically changing tumor microenvironment (TME), ROS played an immunosuppressive participant in tumor progression. The high levels of ROS in the TME made immune cells vulnerable to ROS-induced damage, and tumor cells had evolved many antioxidant defense mechanisms to escape the damage of oxidative stress (16). Thus, the generation of ROS greatly contributed to tumor-induced immunosuppression, which promoted tumor invasion, metastasis, and resistance. However, the prognostic value of ROS-related genes in ccRCC has not been elucidated. Taken together, exploring the role of ROS in the immune landscape of ccRCC would facilitate the prognosis prediction and provide tailored treatment strategies for each individual.

In this study, we aimed to clarify that ROS-related genes had a prognostic effect by investigating the differences in the expression levels between ccRCC and normal tissues. On this basis, we constructed an ROS-related signature as a prognostic biomarker, systematically investigated the role of our signature in immune infiltration, and further provided clinical evidence for guiding immunotherapy and targeted therapy.

## 2 Materials and methods

### 2.1 Datasets

Human ccRCC tissue microarrays (TMA, Wellbio, China, ZL-KIC1601), with detail clinical information comprising of gender, age, tumor size, grade, and tumor node metastasis (TNM) stage, containing 80 ccRCC samples and 80 adjacent benign tissues, were conducted for immunohistochemical (IHC) staining. RNA sequencing data and relevant clinicopathological information of ccRCC samples were retrieved from The Cancer Genome Atlas (TCGA) database (https://tcga-data.nci.nih.gov/tcga/). The caret R package was conducted to randomly divide the entire TCGA dataset into two cohorts: a training cohort and a testing cohort. The training cohort was appointed to develop a signature, whereas the testing cohort was applied to validate it. The E-MTAB-1980 dataset (https://www.ebi.ac.uk/arrayexpress/experiments/E-MTAB-1980/) was extracted as a validation cohort. We obtained 49 ROS-related genes from hallmark gene sets within the GSEA Molecular Signatures Database (MSigDB; http://www.gsea-msigdb.org/gsea/msigdb/cards/HALLMARK_REACTIVE_OXYGEN_SPECIES_PATHWAY.html).

### 2.2 Identification of differentially expressed genes

The limma package was used to identify ROS-related genes with a P-value < 0.05. The Search Tool for Retrieval of Interacting Genes (STRING) was employed to create a protein–protein interaction (PPI) network. The Rcircos R package was utilized to examine the CNV feature present in human chromosomes.

### 2.3 Consensus clustering

To investigate different biological modifications of ROS-related genes in patients with ccRCC, we applied consensus clustering to separate the samples into different patterns *via* the ConsensusClusterPlus package. The optimal number of subtypes was assessed by cumulative distribution function (CDF) and consensus matrices.

### 2.4 Construction and evaluation of the signature

Univariate Cox regression analysis was employed to determine prognostic ROS-related genes. Subsequently, the Lasso, known as the least absolute shrinkage and selection operator, was utilized to build a signature employing 10-fold cross-validation, resulting in a $\text{risk score}=\sum_{\text{i}}^{\lambda }\text{βiSi}$ . The signature’s predictive effectiveness was evaluated through receiver operating characteristic (ROC) curves and Kaplan–Meier analysis. Univariate and multivariate Cox regression analyses were used to reconfirm the independent prognostic value. The R timeROC, survminer, and survival packages were used in these procedures. The nomogram was adopted to predict the 1-, 2-, and 3-year overall survival *via* the rms package.

### 2.5 Gene set enrichment analysis

The clusterProfiler R package was subjected to carry out Kyoto Encyclopedia of Genes and Genomes (KEGG) and Gene Ontology (GO) analyses, encompassing categories such as cellular component (CC), molecular function (MF), and biological process (BP). In addition, the GSEA software version 4.0.3 was employed for gene set enrichment analysis (GSEA).

### 2.6 Immune infiltration analysis

We used Tumor IMmune Estimation Resource (TIMER; https://cistrome.shinyapps.io/timer/), a website that comprehensively analyzes tumor-infiltrating immune cells, to analyze the relationship between ROS-related genes and immune infiltration. The “Gene” module of TIMER allows visualization of the correlation between gene expression and the levels of immune infiltration. The ESTIMATE algorithm was conducted to calculate the ESTIMATE scores, immune scores, stromal scores, and tumor purity. The abundance of immune infiltration was estimated by CIBERSORT, CIBERSORT-ABS, TIMER, XCELL, EPIC, QUANTISEQ, and MCPCOUNTER algorithms. The Wilcoxon signed-rank test was employed to assess the variation in immune infiltrating cells between the both risk groups. The relationship between the signature and immune checkpoints or human leukocyte antigen (HLA) expression was also identified by aforementioned analysis. Moreover, single-sample GSEA (ssGSEA) was used to evaluate immune cell infiltration and immune function in two subgroups. These processes were performed on the basis of the R ggpubr, GSEABase, GSVA, limma, and reshape2 packages.

### 2.7 The sensitivity of targeted therapy and immunotherapy

We initially collected gene expression data of patients with ccRCC from TCGA program using standard procedures. pRRophetic was an R package for predicting drug sensitivity from gene expression levels. This package utilizes a pre-trained model that correlates gene expression data with drug response data from large-scale pharmacogenomics datasets. The median inhibitory concentration (IC50) of targeted drugs, representing the drug concentrations required to inhibit 50% of the cellular response, was calculated on the basis of the pRRophetic R package. The distinction in targeted therapy between the two risk groups was identified by Wilcoxon signed-rank test using the ggplot2 R package. In addition, the CellMiner program, including 60 cancer cell lines in nine different tissues, was utilized to evaluate the relationship between four ROS-related genes and drug sensitivity through Pearson correlation analysis (https://discover.nci.nih.gov/cellminer). The immunophenoscore (IPS) of patients with ccRCC was downloaded from the TCIA (https://tcia.at/), and the tumor immune dysfunction and exclusion (TIDE) was determined by online tool (http://tide.dfci.harvard.edu/), which was positively correlated with immunotherapy.

### 2.8 Immunohistochemical staining

The protein expression levels of glutamate-cysteine ligase modifier subunit (GCLM), methionine sulfoxide reductase A (MsrA), and strawberry notch homolog 2 (SBNO2) were assessed in a total of 80 paired ccRCC and adjacent non-tumor samples by IHC. IHC staining was obtained according to the instruction of the IHC kit (KIT-9730, MX Biotechnologies, Fuzhou, China). The antibodies—SBNO2 (bs-23726R, Bioss, Beijing, China), MsrA (14547-1-AP, Proteintech, Wuhan, China), and GCLM (ET1705-87, Huabio, Hangzhou, China)—were used in IHC staining. The final immunoreactivity score was determined by multiplying the proportion of positively stained regions and intensity score. The staining regions was calculated and classified as: 0 (0), 1 (1%–9%), 2 (10%–50%), 3 (51%–80%), and 4 (81%–100%). The staining intensity was classified as 0 (negative), 1 (weak), 2 (moderate), and 3 (strong).

### 2.9 Statistical analysis

Some related abovementioned R packages were conducted to perform statistical analysis on the basis of R version 4.1.1. SPSS 26 was suitable for Wilcoxon signed-rank test. P-value of < 0.05 was regarded as statistically significant.

## 3 Results

### 3.1 Identification of differentially expressed ROS-related genes in ccRCC

**Figure 1:**
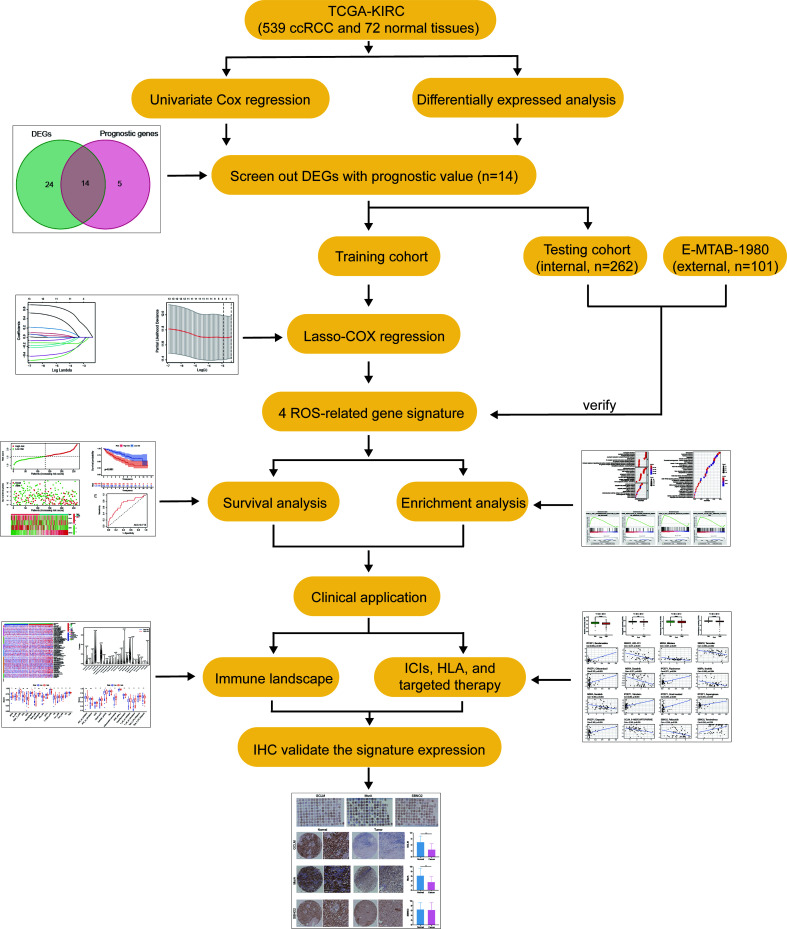
The main workflow in this study.

**Figure 2:**
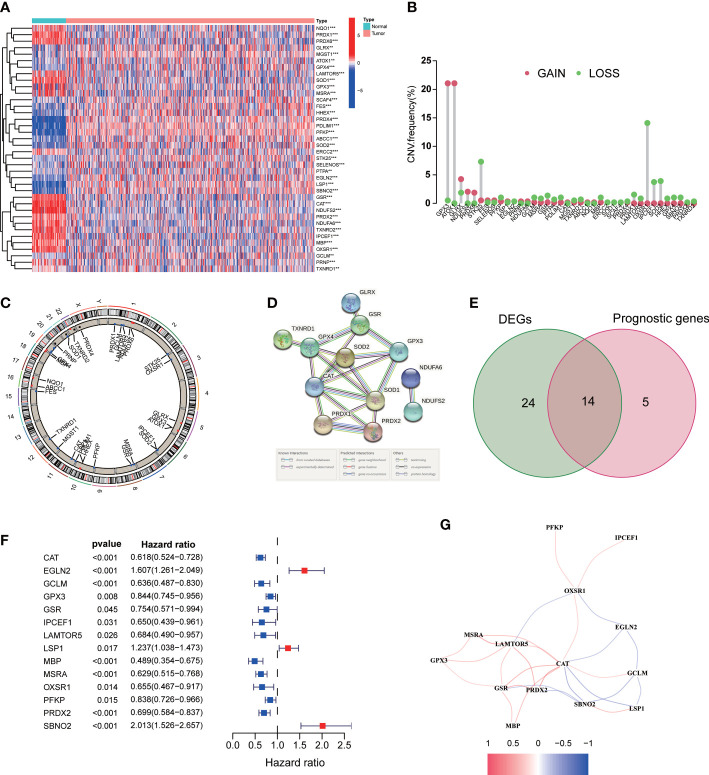
Expression of the ROS-related genes in ccRCC. **(A)** ROS-related DEGs in ccRCC and adjacent benign samples. **(B)** Frequencies of CNV gain and loss among DEGs. **(C)** The location of CNV alteration of DEGs on chromosomes. **(D)** PPI network. **(E)** Venn diagram of 38 DEGs and 19 prognostic genes. **(F)** Univariate Cox regression analysis of 14 prognostic genes. **(G)** The correlation network of 14 DEGs. **p<0.01 and ***p<0.001.

As shown in our workflow diagram in **Figure 1**, we first analyzed the differential expression of ROS-related genes in 539 ccRCC samples and 72 normal tissues from the TCGA database. There were 38 differentially expressed genes (DEGs) with distinct distribution in normal and tumor tissues (**Figure 2A**). The frequency of CNV alterations in DEGs showed that most of them were primarily concentrated on copy number reduction (**Figure 2B**). We performed chromosome annotation to precisely identify the sites of CNV alterations for 38 DEGs (**Figure 2C**). PPI analysis with a minimum interaction score of 0.9 was to explore the interactions among these DEGs (**Figure 2D**). In addition, 19 prognostic genes were detected to be notably associated with the prognosis of ccRCC. After overlapping DEGs and prognostic genes through the Venn diagram, we found that 14 genes were both DEGs and prognostic genes (**Figure 2E**). The univariate Cox regression analysis was performed to reveal that all 14 genes were significant (**Figure 2F**). The correlation network of them was displayed in **Figure 2G**.

### 3.2 Tumor classification based on ROS-related genes

To investigate the association between the expression of ROS-related genes and ccRCC subtypes, patients with ccRCC in TCGA database were grouped into clusters. The empirical CDF was used to determine the optimum k-values for the sample distribution with maximal stability (**Figure S1A**). We found that, when clustering variable (k) = 2, patients with ccRCC could be well divided into two different clusters (**Figure S1B**). However, different clusters failed to show a clear distinction when k = 3 or 4 based on the results of consensus matrix heatmap and survival analysis (**Figures S1C, D**). The distribution of clinical characteristics including survival status and gender differed between the two clusters (**Figure S1E**).

### 3.3 Construction of prognostic signature for ccRCC

**Figure 3:**
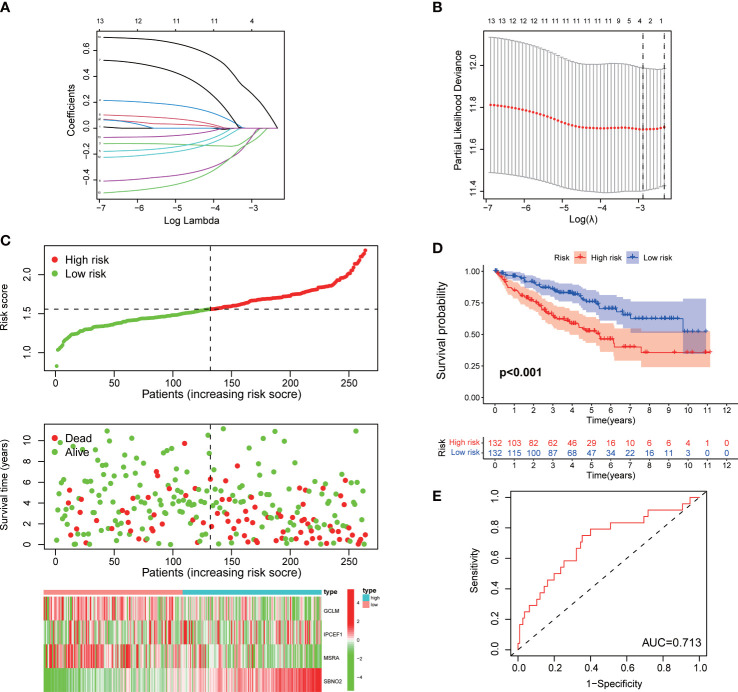
Construction of a signature for ccRCC in the training cohort. **(A, B)** Lasso regression and cross-validation. **(C)** The risk score, survival status, and heatmap of the signature. **(D)** Kaplan–Meier survival curve of the signature. **(E)** ROC curves.

The patients with ccRCC of TCGA were randomly and equally divided into the training and testing cohorts. The training cohort was used to construct a signature for predicting prognosis. We performed univariate Cox regression on 14 DEGs with prognostic value in the training cohort. Furthermore, to avoid overfitting prognostic markers, we performed Lasso regression analysis and identified the optimal penalty parameter values using 10-fold cross-validation (**Figures 3A, B**). Eventually, we identified four effective ROS-related genes for the construction of the risk signature. Patients with ccRCC were divided into a high-risk group and a low-risk group according to the median risk score. As the risk score increased, the mortality rate of patients with ccRCC gradually increased (**Figure 3C**). Meanwhile, patients in the high-risk group suffered a poorer prognosis (**Figure 3D**). We conducted the ROC analysis and determined that the signature exhibited an area under curve (AUC) of 0.713 in the training cohort, indicating a favorable sensitivity and specificity in predicting the prognosis of patients with ccRCC (**Figure 3E**).

### 3.4 Validation of signature in survival prediction for ccRCC

To further validate the ability of the signature to independently predict prognosis, we performed validation in the testing cohort and E-MATB-1980, an independent dataset that served as the external validation cohort. Mortality events of patients with ccRCC in the two cohorts increased with growing risk scores (**Figures S2A, B**). The AUC in the testing and validation cohorts were 0.713 and 0.769, respectively, which exhibited excellent prediction accuracy (**Figures S2C, D**). Furthermore, patients in the high-risk group had a worse prognosis than those in the low-risk group in two ccRCC cohorts, which was consistent with the results of the training cohort (**Figures S2C, D**). The results of univariate and multivariate Cox regression analysis suggested that the signature was an independent factor for overall survival prediction in the three cohorts (**Figure S3**).

### 3.5 Relationship between clinical features and the signature

**Figure 4:**
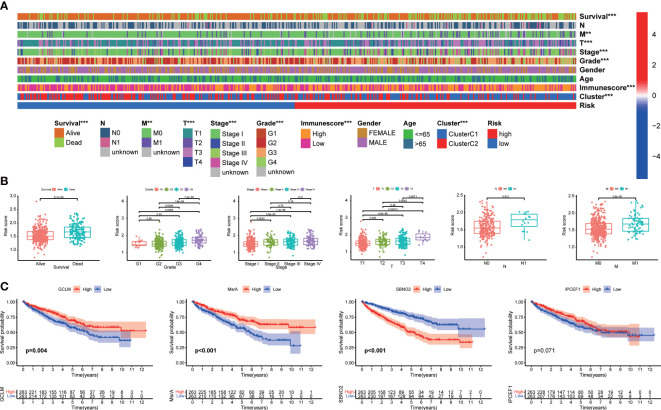
The correlation between the signature and clinical characteristics. **(A)** The distribution of clinicopathological characteristics. **(B)** Risk scores were significantly associated with survival status, grade, TNM stage, T stage, N stage, and M stage. **(C)** Kaplan–Meier analysis of four signature genes.

As illustrated in the heatmap, the survival status, M stage, T stage, TNM stage, grade, immunescore, and clusters were diversely distributed in the two groups (**Figure 4A**). Our signature was closely associated with the clinicopathological characteristics including survival status, grade, TNM stage, T stage, N stage, and M stage (**Figure 4B**). The high-risk group was more likely to be patients with high-grade and advanced stage. Kaplan–Meier analysis revealed that a high expression of GCLM and MsrA predicted a favorable prognosis, whereas SNBO2 showed the opposite trend (**Figure 4C**). There was no significance for survival outcomes in the expression of interaction protein for cytohesin exchange factors 1 (IPCEF1) (**Figure 4C**). Dividing patients with ccRCC into distinct stratification groups based on age, gender, grade, and TNM stage, we found that the high-risk group all represented a worse prognosis in the stratification subgroups (**Figures S4A–F**). Consequently, notably correlated to the prognosis and progression of ccRCC, our signature had a broad applicability and feasibility in prognostic prediction.

### 3.6 Construction of the nomogram and distribution patterns

**Figure 5:**
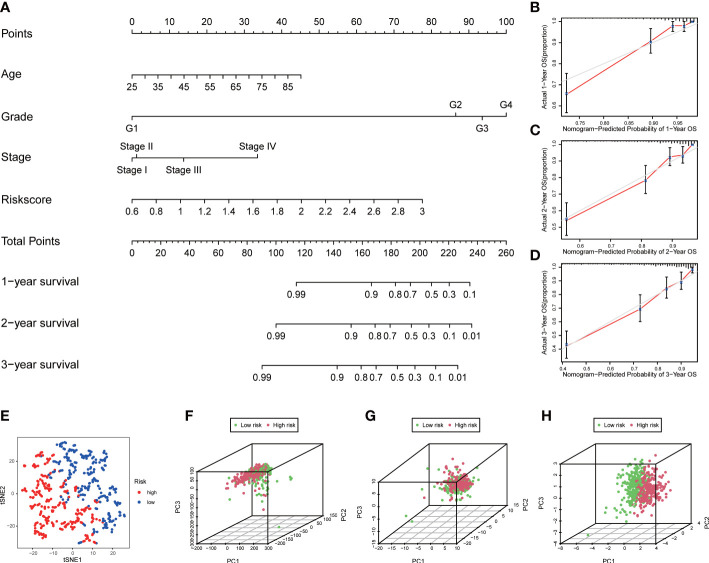
Construction of nomogram and distribution patterns. **(A)** The nomogram predicted the 1-, 2-, and 3-year overall survival rates. **(B–D)** Calibration curves for the nomogram. **(E)** t-SNE analysis. 3D PCA between the low- and high-risk groups based on **(F)** genome-wide expression profiles, **(G)** all ROS-related genes, and **(H)** four ROS-related genes.

A nomogram containing risk scores and clinical characteristics was constructed to predict the probability of survival for patients with ccRCC at 1, 2, and 3 years (**Figure 5A**). We then demonstrated the consistency of our nomogram’s observation and prediction at 1, 2, and 3 years by calibration charts (**Figures 5B–D**). The t-distributed Stochastic Neighbor Embedding (t-SNE) showed that patients in the two groups were well separated into two clusters (**Figure 5E**). The principal component analysis (PCA) revealed that the two groups did not show a clear separation based on genome-wide expression profiles and all ROS-related genes (**Figures 5F, G**). However, patients could be divided into two distinct directions on the basis of the signature of four ROS-related genes (**Figure 5H**).

### 3.7 Functional enrichment analyses

**Figure 6:**
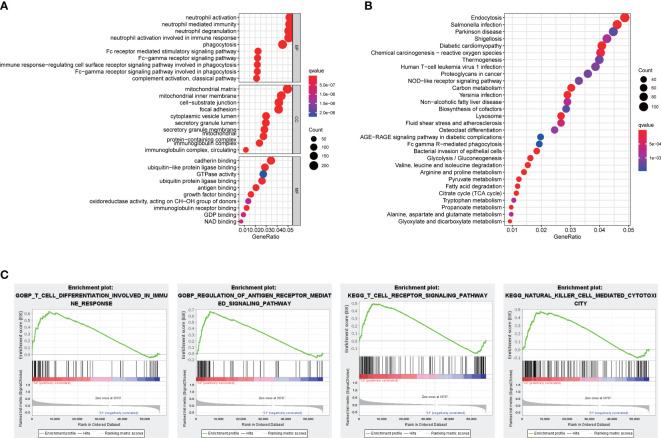
Functional analyses. **(A)** Bubble graph for GO enrichment and **(B)** KEGG pathways. **(C)** Enrichment plot by GSEA analysis.

To gain deeper insights into the BPs and potential molecular mechanisms related to the signature, we undertook the analyses of GO and KEGG, revealing the participation of many immune-related BPs (**Figures 6A, B**). Moreover, functional annotation was further validated by GSEA, and the results suggested that immune responses including T-cell differentiation involved in immune response, regulation of antigen receptor–mediated signaling pathway, T-cell receptor signaling pathway, and natural killer cell mediated cytotoxicity were further enriched in the high-risk groups compared to the low-risk groups (**Figure 6C**).

### 3.8 The landscape of immune cell infiltration and immune function in ccRCC

**Figure 7:**
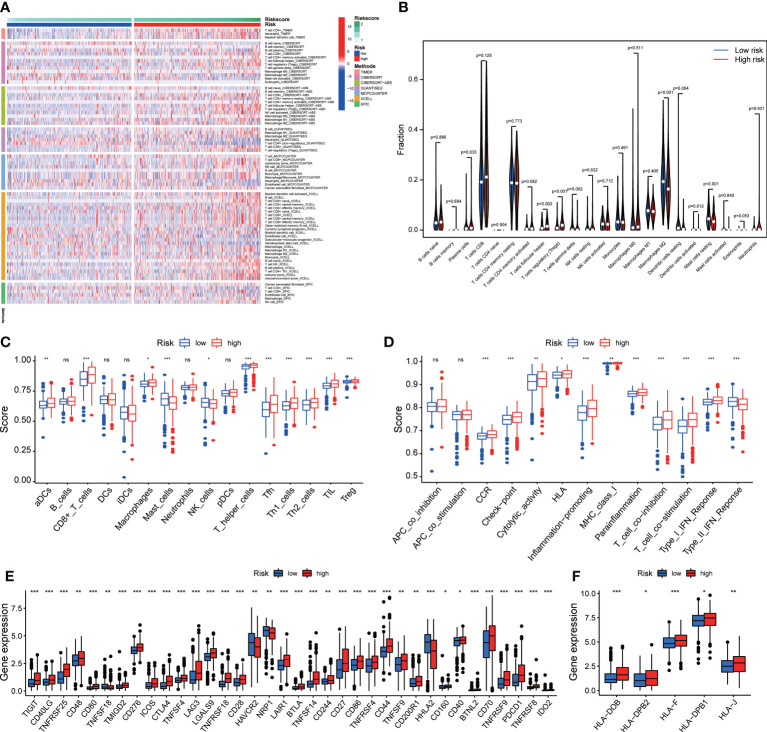
The immune cell infiltration and immune function. **(A)** The difference between the signature and the abundance of immune cells in seven algorithms. **(B)** The relationship between the signature and immune cells according to CIBERSORT. **(C)** Immune cell infiltration and **(D)** immune function by ssGSEA algorithm. The differences in the expression of **(E)** immune checkpoint and **(F)** HLA family. *p<0.05, **p<0.01, and ***p<0.001. ns, not significant.

We analyzed the relationship between immune cells and the four ROS-related genes, which showed that they were all positively associated with B cells, CD8+ T cells, CD4+ T cells, macrophages, neutrophils, and dendritic cells (DCs) (**Figures S5A–D**). To better understand the tumor immune status of ccRCC, the ESTIMATE algorithm was applied to calculate the stromal score, immune score, ESTIMATE score, and tumor purity (**Figures S6A, B** **)**. The high-risk group had a higher ESTIMATE score but lower tumor purity. Given the above results, multiple algorithms revealed that the signature was closely related to multiple immune cells (**Figure 7A**). Compared with the low-risk group, the abundance of infiltrating CD8 + T cells and regulatory T cells (Tregs) in the high-risk group was significantly higher according to CIBERSORT data (**Figures 7A, B**). Performing the ssGSEA algorithm to evaluate immune cell infiltration yielded similar results (**Figure 7C**). Subsequently, the immune function suggested that CCR, immune checkpoints, HLA, and MHC class I exhibited significant differences between the two groups (**Figure 7D**). The high-risk group was significantly correlated with higher expression of T cell immune receptor with Ig and ITIM domains (TIGIT), cytotoxic T lymphocyte-associated antigen-4 (CTLA-4), lymphocyte activation gene-3 (LAG3), and PD-1 than the low-risk group (**Figure 7E**). The expression of HLA genes in the high-risk group was also higher than that in the low-risk group (**Figure 7F**). In addition, IPS and TIDE were used to assess the sensitivity of patients with ccRCC to ICIs (**Figures S7A–C**). The results of two analysis methods both indicated that the high-risk group obtained a favorable immunotherapeutic response and effect. The specificity and sensitivity of our signature were superior to that of recently identified biomarkers such as TIS and TIDE (**Figure S7D**).

### 3.9 The sensitivity analysis of targeted therapy and chemotherapy in ccRCC

**Figure 8:**
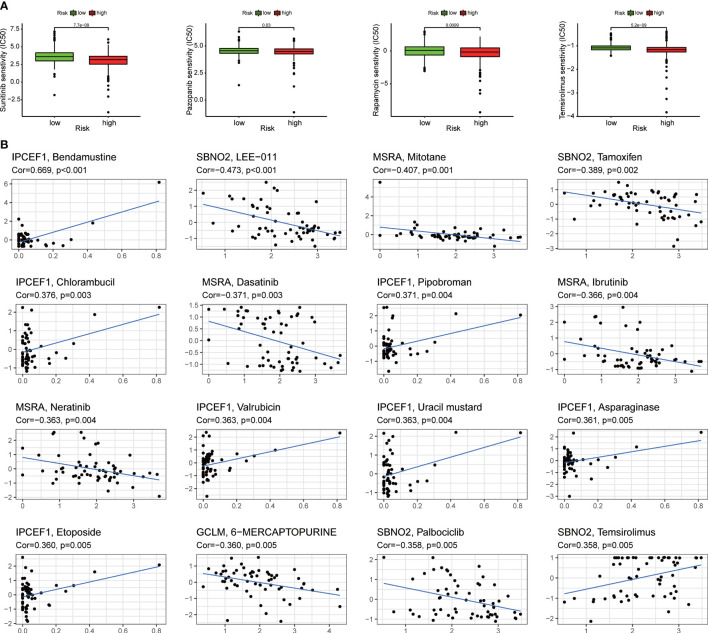
Assessing the sensitivity of targeted therapy. **(A)** Patients with ccRCC in the high-risk group were suitable for targeted drugs such as sunitinib, pazopanib, rapamycin, and temsirolimus. **(B)** The relation between different drugs and four ROS-related genes.

We assessed the responsiveness of distinct risk groups to targeted medications and chemotherapy in the ccRCC data of the TCGA project. The results suggested that sunitinib and pazopanib targeting multiple tyrosine kinase targets and mechanistic target of rapamycin (mTOR) inhibitors such as rapamycin and temsirolimus had a lower semi-inhibition rate (IC50) in the high-risk group, indicating that clinicians should be more likely to give targeted drugs to patients in the high-risk group to achieve better outcomes during treatments (**Figure 8A**). In addition, the analysis of NCI-60 panel revealed that each of the four ROS-related genes was also significantly associated with different drugs (**Figure 8B**).

### 3.10 Experimental verification of GCLM, MsrA, and SBNO2 in our patients with ccRCC

**Figure 9:**
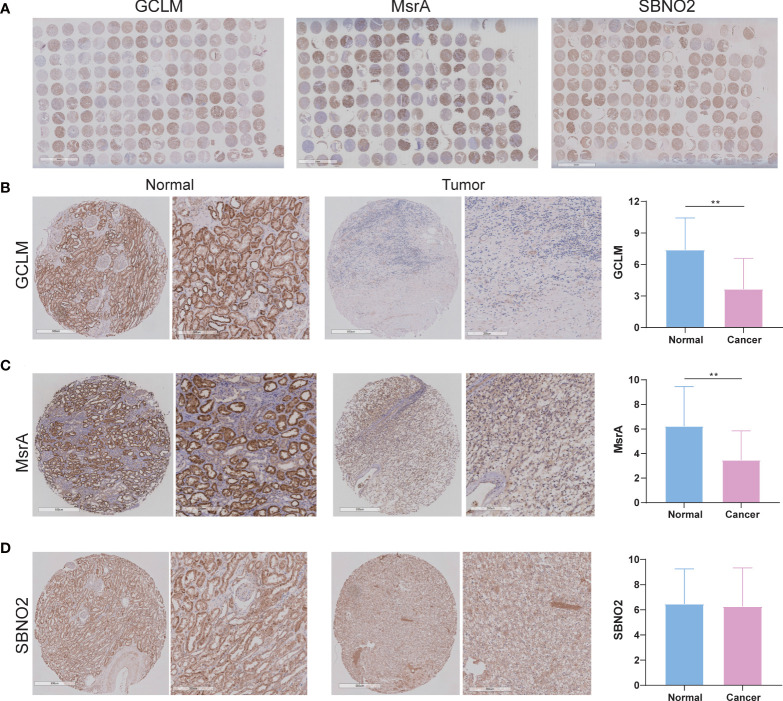
The expression of GCLM, MsrA, and SBNO2 between ccRCC and adjacent benign tissues. **(A)** The overview of IHC staining for GCLM, MsrA, and SBNO2. **(B–D)** The IHC staining of GCLM, MsrA, and SBNO2 in ccRCC and normal tissues. **p<0.01.

Given that survival analysis of GCLM, MsrA, and SBNO2 showed significantly different, we selected them for the next investigation. In addition, GCLM is closely associated with the development of kidney cancer, and the ability of MsrA is to protect the kidney against ischemia-reperfusion injury (17, 18). However, whether they were closely associated with ccRCC was not yet clear. The overview of IHC staining for GCLM, MsrA, and SBNO2 was depicted in **Figure 9A**. IHC staining of ccRCC TMA revealed that GCLM and MsrA were significantly decreased in tumor tissues, whereas SBNO2 showed no significantly different (**Figures 9B–D**). **Table 1** summarized the relation of GCLM, MsrA, and SBNO2 expression to clinical features in our patients with ccRCC.

**Table 1:** The relation of GCLM, MsrA, and SBNO2 expression to clinical features in our patients with ccRCC.

Clinical features	GCLM			MsrA			SBNO2		
	Case	x ± s	*P*	Case	x ± s	*P*	Case	x ± s	*P*
Tissue									
Normal	80	7.39 ± 3.05	**<0.001**	69^#^	6.23 ± 3.22	**<0.001**	69^#^	6.46 ± 2.78	0.540
Cancer	80	3.66 ± 2.92		79^##^	3.47 ± 2.38		79^##^	6.27 ± 3.06	
Gender									
Male	54	3.30 ± 2.53	0.258	53	3.43 ± 2.38	0.804	53	6.17 ± 3.17	0.633
Female	26	4.42 ± 3.54		26	3.54 ± 2.42		26	6.46 ± 2.87	
Age									
≤60	28	4.04 ± 2.99	0.804	27	3.44 ± 1.97	0.980	27	5.70 ± 2.76	0.325
>60	17	3.88 ± 3.16		17	3.65 ± 2.52		17	6.82 ± 3.11	
Tumor size									
≤4	46	3.37 ± 2.58	0.513	46	3.09 ± 2.25	0.088	46	5.93 ± 3.14	0.267
>4	34	4.06 ± 3.33		33	4.00 ± 2.49		33	6.73 ± 2.92	
Grade									
I	30	2.87 ± 2.27	0.272	29	4.24 ± 2.34	0.065	29	6.79 ± 3.05	0.152
II	40	4.13 ± 3.20		40	2.88 ± 2.19		40	6.33 ± 3.12	
III	10	4.20 ± 3.26		10	3.60 ± 2.80		10	4.50 ± 2.32	
TNM stage									
I	72	3.36 ± 2.72	**0.030**	71	3.23 ± 2.24	**0.043**	71	6.06 ± 3.03	0.175
II	6	7.33 ± 3.45		6	6.17 ± 2.64		6	8.50 ± 3.21	
III	2	3.50 ± 0.71		2	4.00 ± 2.83		2	7.00 ± 1.41	
T stage									
T1a	46	3.37 ± 2.58	0.122	46	3.09 ± 2.25	0.128	46	5.93 ± 3.14	0.277
T1b	26	3.35 ± 3.02		25	3.48 ± 2.24		25	6.28 ± 2.85	
T2a	4	7.25 ± 4.27		4	6.50 ± 2.65		4	7.50 ± 3.42	
T2b	2	7.50 ± 2.12		2	5.50 ± 3.54		2	10.5 ± 2.12	
T3a	2	3.50 ± 0.71		2	4.00 ± 2.83		2	7.00 ± 1.41	
N stage									
N0	80	3.66 ± 2.92	–	79	3.47 ± 2.38	–	79	6.27 ± 3.06	–
N1	0	–		0	–		0	–	
M stage									
M0	80	3.66 ± 2.92	–	79	3.47 ± 2.38	–	79	6.27 ± 3.06	–
M1	0	–		0	–		0	–	

# 11 cases dropped off, ## 1 case dropped off. Bold values indicate statistical significance (p<0.05)

## 4 Discussion

ccRCC is the most common subtype of kidney cancer, and its incidence ranks third among urinary system cancers (1). Early diagnosis and timely surgical resection are urgent measures to improve the therapeutic effect of ccRCC (19). However, for patients with advanced stage or distant metastasis who could lose an opportunity to have surgery, their 5-year survival rate was only 12% (20). For some patients with ccRCC with similar clinical manifestations, because of the heterogeneity of tumor and the diversity of biomolecules, their therapeutic effects and clinical prognosis were also different (21, 22). Therefore, it was suggested that existing predictors of ccRCC prognosis were inadequate to meet current clinical needs, and we must identify a new and more accurate signature.

In this study, we constructed, validated, and evaluated a signature composed of GCLM, IPCEF1, MsrA, and SBNO2, which effectively predicted the prognosis and was involved in immune-related pathways in patients with ccRCC. GCLM constituted the first rate-limiting enzyme for glutathione synthesis. GCLM was upregulated in a variety of human tumor types, and patients with the high level of GCLM mRNA had lower recurrence-free and overall survival rates. Moreover, genetic deletion of GCLM prevented the ability of tumor to drive malignant transformation (23). As for IPCEF1, which was related to peroxidase activity and oxygen transporter activity, participated in ADP-ribosylation factor 6 signaling events and oxidative stress (24). The latest research showed that enforced expression of IPCEF1 inhibited the migration potential of T helper cell 17 (Th17) cells (25). MsrA reduced intracellular ROS levels through circulating oxidative/reductive mechanisms, and overexpression of MsrA enhanced cellular resistance to oxidative stress and protection against damage (26). As for SBNO2, a component of the IL-10 signaling cascade in monocytes, inhibited nuclear factor kappa-B (NF-κB) signaling pathway in macrophages (27). Our signature was associated with tumor immune response and oxidative stress, providing a novel approach to elucidate the prognosis prediction and treatment guidance in ccRCC.

The tumor immune microenvironment played a crucial role in ccRCC, which was one of the tumors with the highest degree of immune infiltration among pan-carcinomas, and its pathological specimens often contain a large number of tumor-infiltrating lymphocytes (9, 28). T cells were a major source of ROS in the TME. Compared with healthy subjects, peripheral blood T cells from patients with systemic sclerosis showed increased ROS production (29). A small amount of ROS could stimulate T cells activation and proliferation, but the accumulation of ROS could induce T-cell apoptosis and functional inhibition (30). Tregs were the key immunosuppressive cells that were increased in patients with cancer. To a certain extent, the levels of ROS determined the function of Tregs (31). It has been recognized that targeted therapy and immunotherapy were also affected by immune cells in the TME (32). Therefore, we analyzed the relationship between the signature and immune cells and found that the high-risk group was positively related to CD8+ T cell and Tregs. Giraldo et al. (33) evaluated IHC samples from 135 patients with ccRCC and found that a high abundance of CD8+ T-cell infiltration was closely related to the poor prognosis. Tregs were lymphocytes that inhibited anti-tumor response, and it had been shown that the increase of Tregs in the TME was related to worse pathological grade and clinical stage in ccRCC (34). Consistent with previous results, the high-risk group patients predicted a worse overall survival rate and exhibited higher levels of immune cells, specifically for CD8+ T cells and Tregs. Combining T-cell–based therapies with antioxidant therapies was a promising therapeutic strategy, emphasizing the significance of immune cell infiltration in ccRCC treatment and clinical outcomes.

Recently, ICIs have proven to be highly effective and are now considered as the standard care for patients with treatment-naive and advanced ccRCC (11). However, there were still a significant proportion of patients who did not benefit from ICIs, which prompted us to further explore the relationship between the expression of immune checkpoints and ccRCC (35). As shown in our results, the expression levels of immune checkpoints were different in separate groups. The immune checkpoints including TIGIT, CTLA-4, LAG3, and PD-1 were highly expressed in the high-risk group. Braun et al. (36) identified that, compared to the normal renal samples and early ccRCC, a higher proportion of M2-like tumor-associated macrophages (TAMs) expressing ligands for multiple T-cell inhibitory receptors such as PD-1, CTLA-4, and TIGIT were enriched in advanced and metastatic ccRCC and were related to a worse prognosis. Consistent with previous results, we analyzed the relationship between the signature and the immune checkpoints and found that the immune checkpoints related to a poor prognosis in ccRCC were highly expressed in the high-risk group, which coincided with the demonstration that our signature could predict overall survival by immunity. Some immune checkpoints might be responsible for a poorer prognosis in the high-risk group providing a new perspective on understanding ccRCC. In addition, the differential expression of immune checkpoints in different risk groups reminded us that this signature could be used to screen different patients to give them appropriate immunotherapy, which might be beneficial in addressing the problem of clinical patients’ insensitivity to immunotherapy. On the basis of this idea, we further analyzed the IPS and TIDE of the two risk groups, and the results showed that the high-risk group was more sensitive to PD-1 and CTLA-4 inhibitors. Therefore, our signature could effectively and specifically stratify the risk of patients with ccRCC, thereby dividing the subgroups of patients who would benefit more from immunotherapy.

The emergence of various targeted therapies has improved the overall survival rate of patients with advanced ccRCC in the past 15 years (2). For a long time, people have recognized that targeted therapy would be affected by immune infiltration in the TME (37). Sunitinib was one of the most used therapeutic drugs in patients with RCC, and it was related to impaired T-cell activation and proliferation *in vitro* and reduced the accumulation of myeloid-derived suppressor cells in the tumor compartment (38). Immunosuppressive cells such as TAM, neutrophils, and DCs could produce vascular endothelial growth factor (VEGF)-related pro-angiogenic cytokines to weaken the effect of targeted anti-angiogenic agents (39). Targeted therapy and immunity were inextricably linked. Our results showed that the high-risk group was more sensitive to the targeted drugs such as sunitinib, pazopanib, rapamycin, and temsirolimus. This meant that we might be able to use this signature to screen out specific patients that were more sensitive to targeted drugs, which, in turn, would guide the clinical use of drugs. The latest result of an ongoing large randomized controlled trial (NCT02684006) proved that, compared with sunitinib alone, patients with advanced ccRCC receiving avelumab, a new kind of PD-L1 inhibitor, combined with axitinib had a significantly longer progression-free survival time (40, 41). Many clinical adverse events occurred in preclinical studies (41, 42). Therefore, we need to be very careful in selecting paired immunotherapy and targeted therapy based on mechanism and preclinical trials. We might be able to use our signature to select the most suitable immunotherapy and targeted therapy for a specific patient from the perspective of immunity or even combine two drugs in synergistic treatments to achieve the best results with the most suitable and least immunotherapeutic and targeted drugs.

## 5 Conclusions

In summary, our signature was a robust and independent factor for ccRCC, which helped predict patients’ survival and prognosis. Our signature was expected to provide a new solution for the clinical decision-making of immunotherapy and targeted therapy for patients with ccRCC.

## Data availability statement

The original contributions presented in the study are included in the article/**Supplementary Material**. Further inquiries can be directed to the corresponding authors.

## Ethics statement

Written informed consent was obtained from the individual(s) for the publication of any potentially identifiable images or data included in this article.

## Author contributions

WZ and XZ contributed to the study conception and design. Material preparation, data collection, and statistical analysis were performed by MC, XL, JY, SC, YD, JL, and HH. The manuscript was written by HL, YL, SZ, and JT. All authors contributed to the article and approved the submitted version.

## Supplementary material

Supplementary Figure 1 Clinical characteristics of ccRCC classification. **(A)** CDF curves and relative changes in the AUC. **(B-D)** Consensus matrix heatmap and survival analysis in different clusters (k = 2, 3, and 4). **(E)** Differences in clinical characteristics between the two clusters.

Supplementary Figure 2 Validation of the signature in internal and external cohorts. Distribution of the risk score, survival status, and heatmap of patients with ccRCC in the **(A)** testing cohort and **(B)** validation cohort. ROC curves analysis and survival curves in the **(C)** testing and **(D)** validation cohorts.

Supplementary Figure 3 The analysis of independently predictive ability. Univariate and multivariate Cox regression analyses in the **(A)** training, **(B)** testing, and **(C)** validation cohorts.

Supplementary Figure 4 Stratification survival analyses. **(A-F)** Survival analysis of subgroups stratified by multiple clinical factors.

Supplementary Figure 5 The relation between four ROS-related genes and immune cell infiltration. The association between various immune cells and the expression of **(A)** GCLM, **(B)** IPCEF1, **(C)** MsrA, and **(D)** SBNO2.

Supplementary Figure 6 The role of the signature in the TME. **(A)** Distribution of tumor purity, ESTIMATE score, immune score, and stromal score. **(B)** The statistical differences in immune score, stromal score, ESTIMATE score, and tumor purity.

Supplementary Figure 7 The IPS and TIDE analysis. **(A, B)** Sensitivity analysis to CTLA-4 and PD-1 by IPS analysis. **(C)** TIDE in the two risk groups. **(D)** ROC curves of signature, TIDE, and TIS.
