# Supplementary figures and images for: A reactive oxygen species–related signature to predict prognosis and aid immunotherapy in clear cell renal cell carcinoma
Source: Front Oncol. 2023 Jul 11;13:1202151. doi: 10.3389/fonc.2023.1202151 (PMC10367095; doi:10.3389/fonc.2023.1202151)

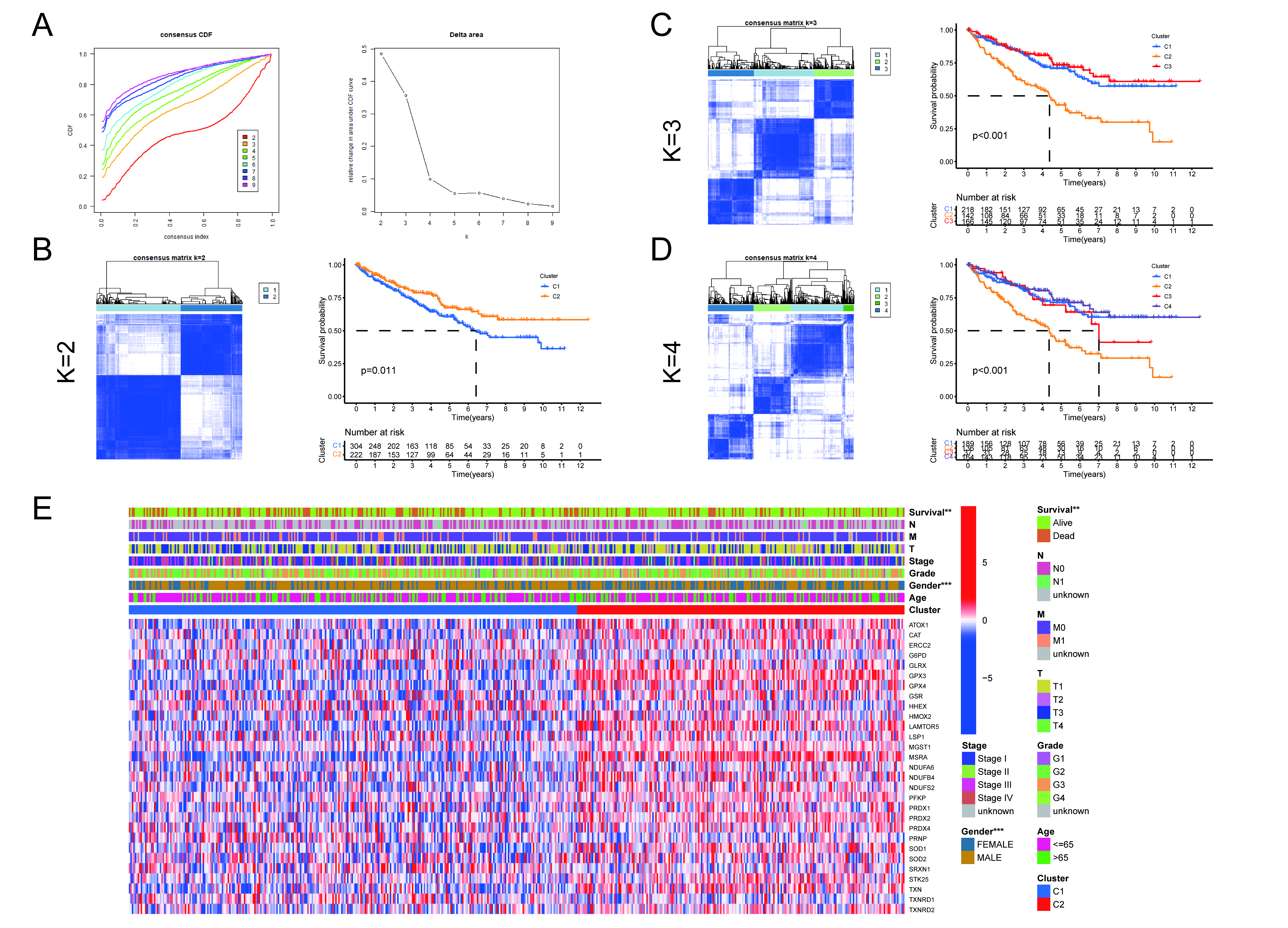

Supplement: Supplementary Figure 1 — Clinical characteristics of ccRCC classification. (A) CDF curves and relative changes in the AUC. (B-D) Consensus matrix heatmap and survival analysis in different clusters (k = 2, 3, and 4). (E) Differences in clinical characteristics between the two clusters. [file Image_1.tif]

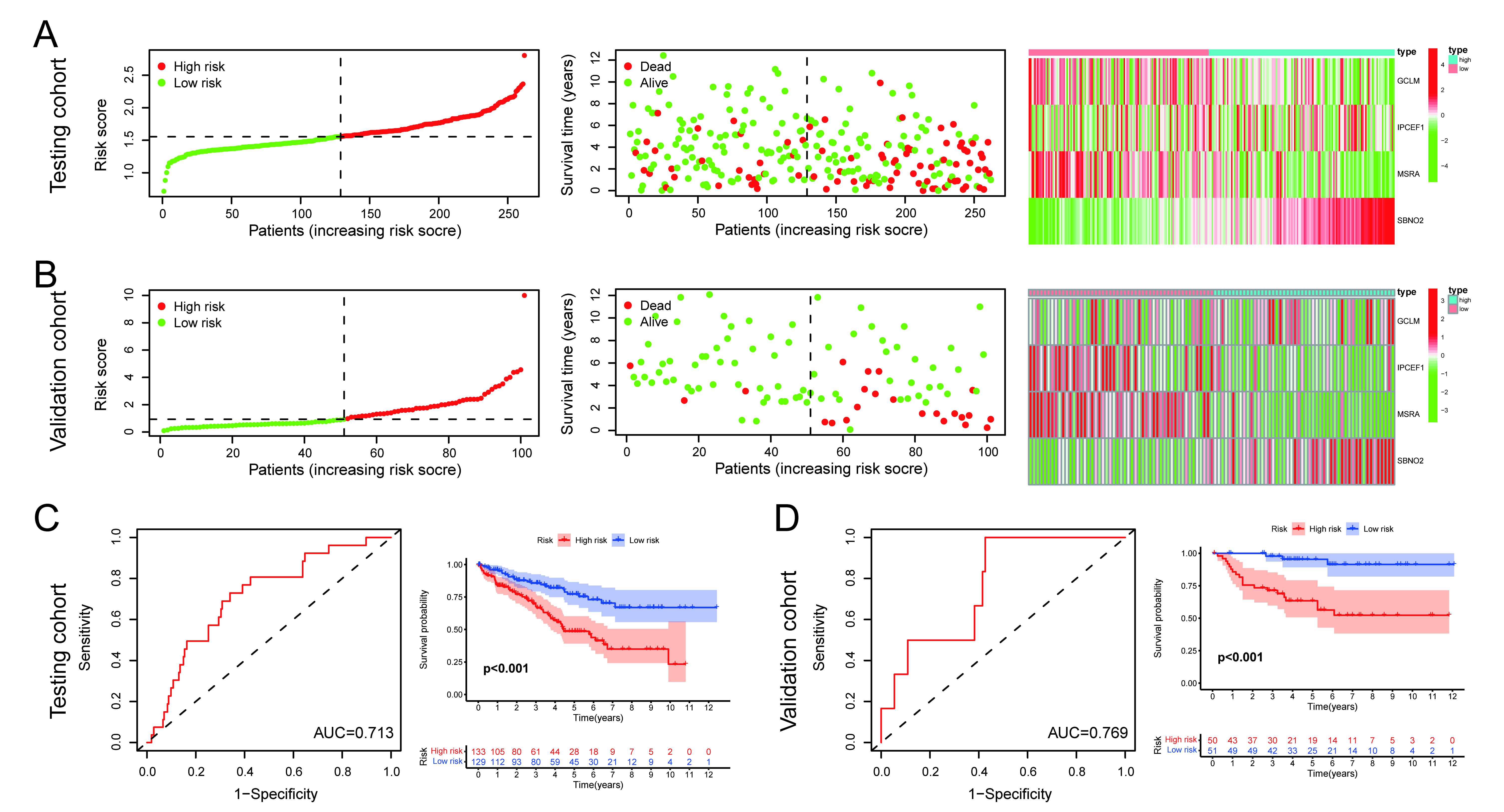

Supplement: Supplementary Figure 2 — Validation of the signature in internal and external cohorts. Distribution of the risk score, survival status, and heatmap of patients with ccRCC in the (A) testing cohort and (B) validation cohort. ROC curves analysis and survival curves in the (C) testing and (D) validation cohorts. [file Image_2.tif]

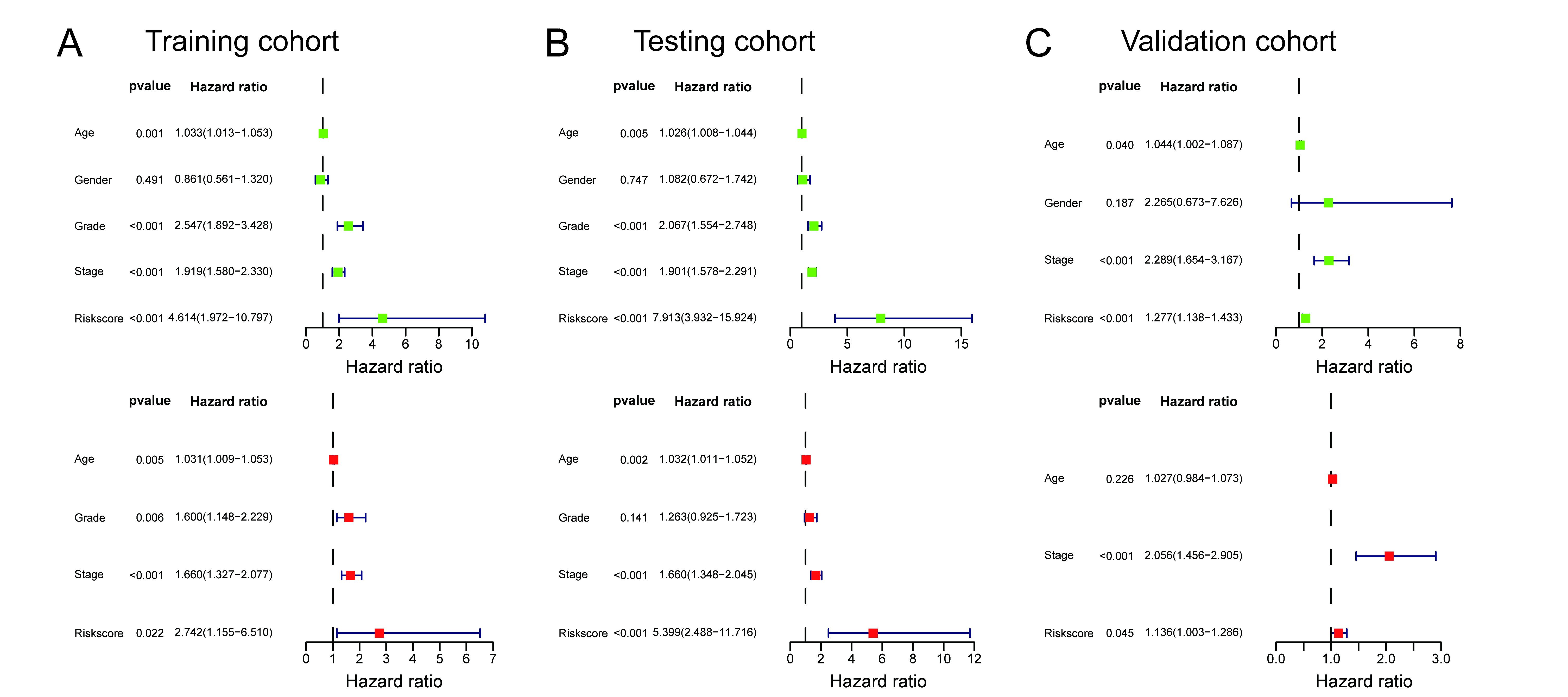

Supplement: Supplementary Figure 3 — The analysis of independently predictive ability. Univariate and multivariate Cox regression analyses in the (A) training, (B) testing, and (C) validation cohorts. [file Image_3.tif]

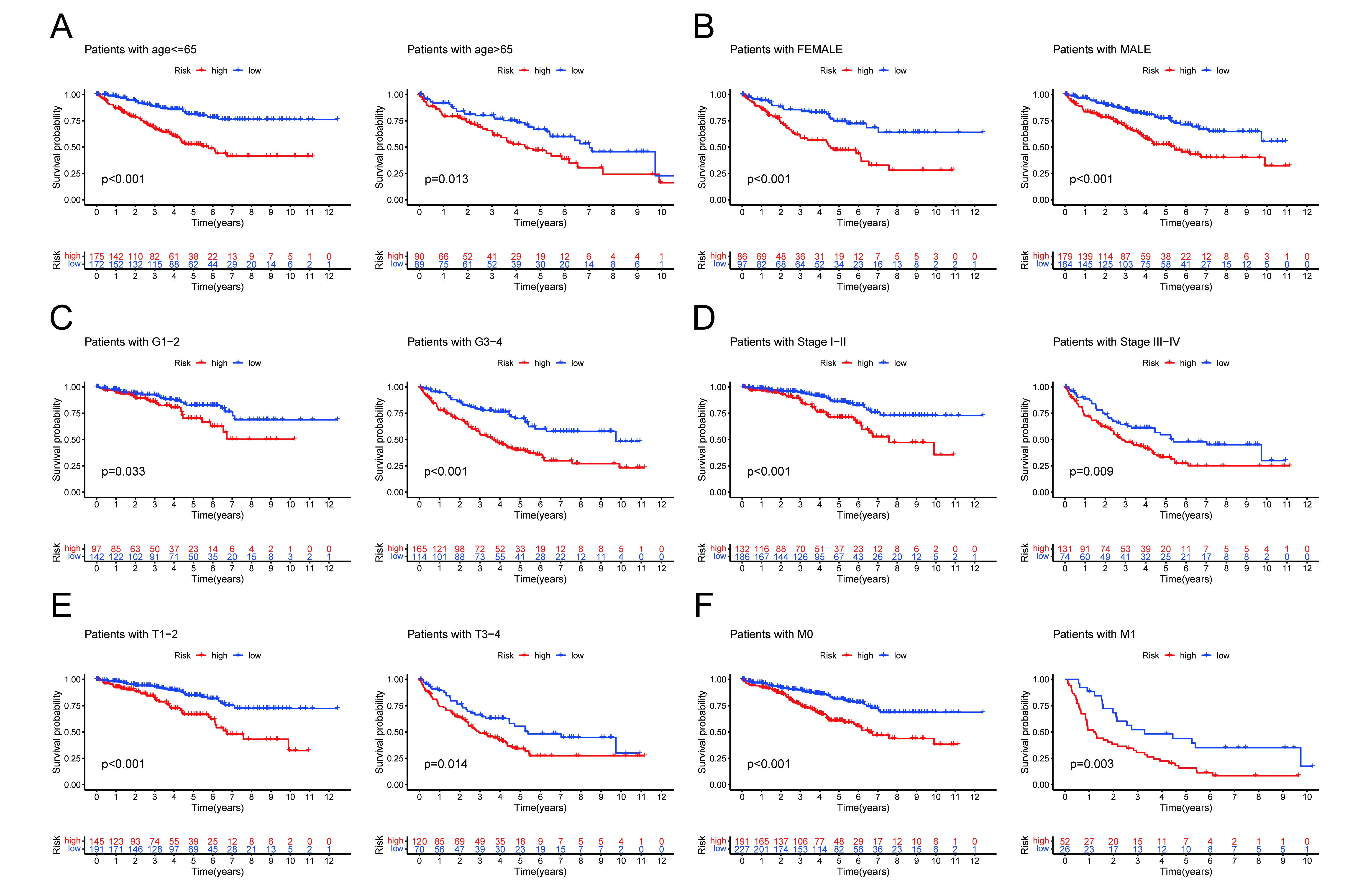

Supplement: Supplementary Figure 4 — Stratification survival analyses. (A-F) Survival analysis of subgroups stratified by multiple clinical factors. [file Image_4.tif]

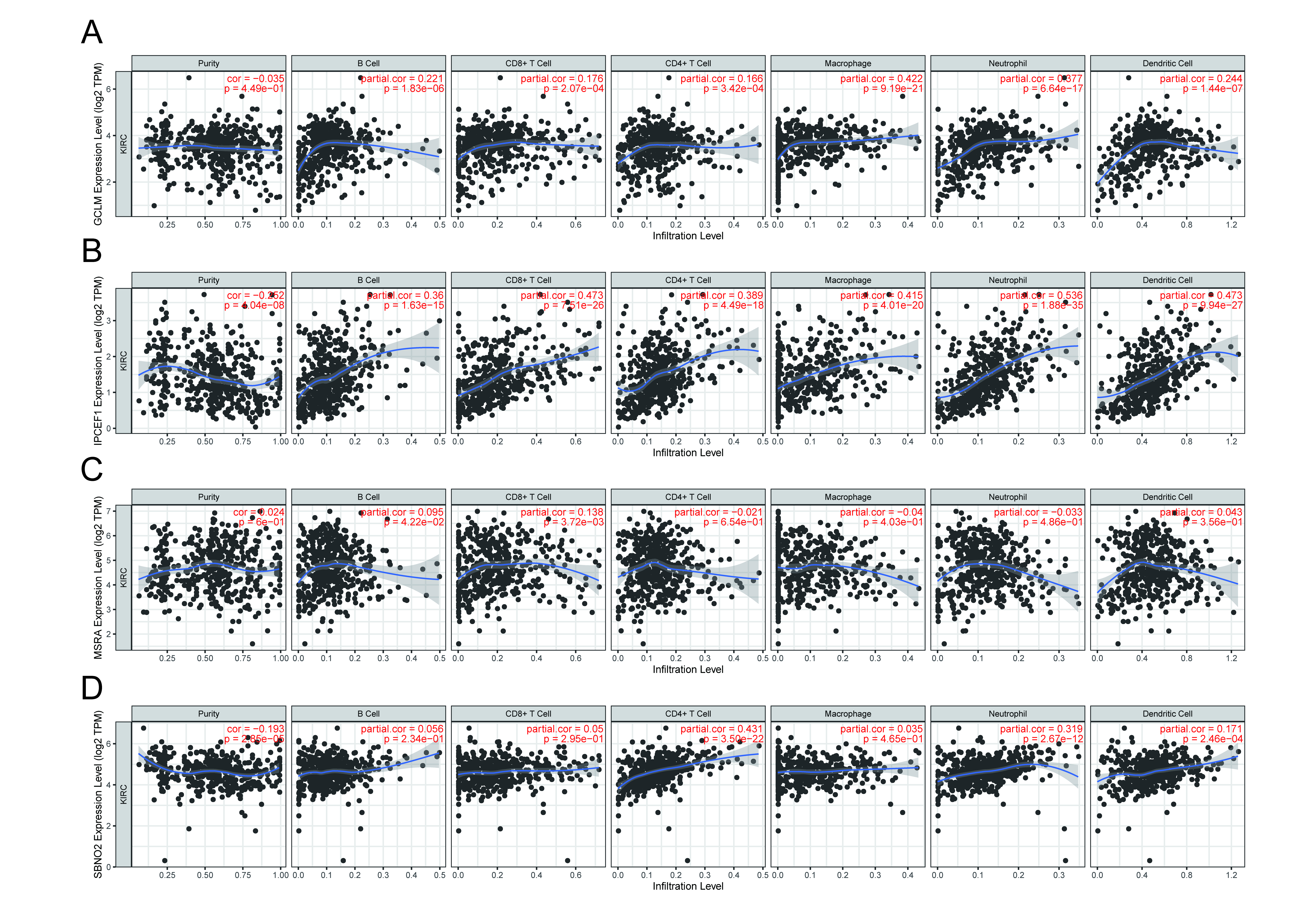

Supplement: Supplementary Figure 5 — The relation between four ROS-related genes and immune cell infiltration. The association between various immune cells and the expression of (A) GCLM, (B) IPCEF1, (C) MsrA, and (D) SBNO2. [file Image_5.tif]

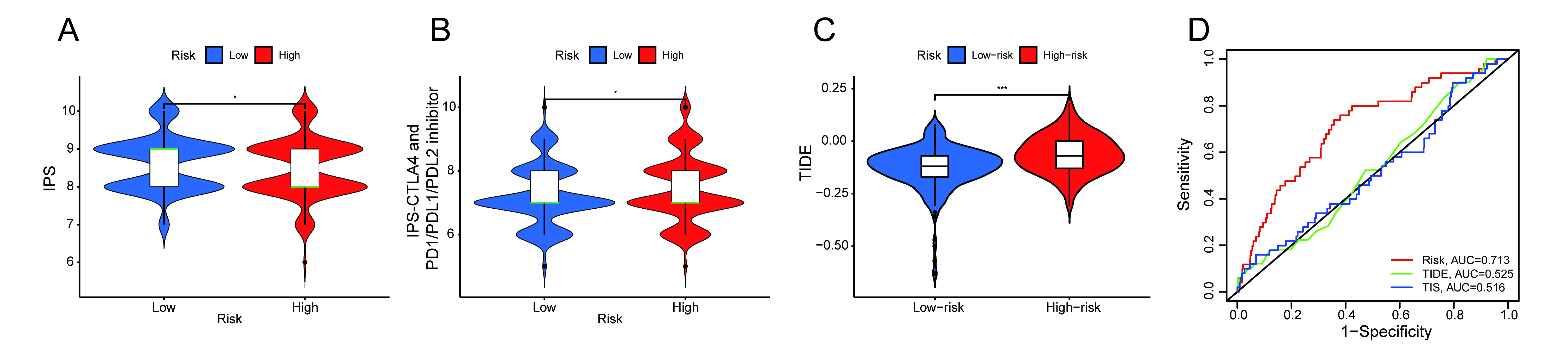

Supplement: Supplementary Figure 7 — The IPS and TIDE analysis. (A, B) Sensitivity analysis to CTLA-4 and PD-1 by IPS analysis. (C) TIDE in the two risk groups. (D) ROC curves of signature, TIDE, and TIS. [file Image_7.tif]
